# CRISPR/Cas system for yeast genome engineering: advances and applications

**DOI:** 10.1093/femsyr/fox030

**Published:** 2017-05-15

**Authors:** Vratislav Stovicek, Carina Holkenbrink, Irina Borodina

**Affiliations:** The Novo Nordisk Foundation Center for Biosustainability, Technical University of Denmark, 2800 Kgs. Lyngby, Denmark

**Keywords:** CRISPR/Cas, CRISPR interference, genome editing, yeasts, CRISPR transcriptional regulation, *Saccharomyces cerevisiae*

## Abstract

The methods based on the clustered regularly interspaced short palindromic repeats (CRISPR)/CRISPR-associated (Cas) system have quickly gained popularity for genome editing and transcriptional regulation in many organisms, including yeast. This review aims to provide a comprehensive overview of CRISPR application for different yeast species: from basic principles and genetic design to applications.

## Teaser

A comprehensive review on application of CRISPR technology in yeast.

## INTRODUCTION

In 2003, Francisco Mojica and colleagues discovered that the spacer sequences from bacterial clustered regularly interspaced short palindromic repeats (CRISPR) loci match viral and conjugative plasmid sequences and hypothesized that CRISPR must be part of the bacterial immune system (Mojica *et al.*^2005^; Lander 2016). In the following years, multiple studies had been performed to unravel the mechanism of CRISPR functionality (Lander 2016) until, in 2012, two research groups managed to reprogram the targeting of CRISPR-associated nuclease (Cas9), so Cas9 would introduce double-strand DNA breaks (DSBs) in a sequence-specific manner *in vitro* (Gasiunas *et al.*^2012^; Jinek *et al.*^2012^). Following this, applications of CRISPR/Cas9 for *in vivo* genome editing in mammalian cells were published early in 2013 (Cong *et al.*^2013^; Mali *et al.*^2013^), followed by DiCarlo *et al.* (2013) reporting the usage of the system in the yeast *Saccharomyces cerevisiae*. Since then the technology has been optimized and adapted for numerous organisms, covering applications from industrial biotechnology (van Erp *et al.*^2015^) to plant breeding (Bortesi and Fischer 2015) and treatment of human diseases (Cai *et al.*^2016^).

In native type II CRISPR/Cas systems, Cas9 is guided to the target DNA region by a two-RNA molecule hybrid consisting of CRISPR RNA (crRNA) and transactivating crRNA (tracrRNA). Together with the tracrRNA, crRNA forms a secondary structure loop, which recruits Cas9. The crRNA guides the system to a genomic target of ∼20 bp through base pairing with the complementary DNA strand. The particular genomic target must be followed by the protospacer adjacent motif (PAM) NGG. The Cas9 nuclease domain HNH then cleaves the DNA-strand complementary to the crRNA-guide sequence, while RuvC-like domain cleaves the other DNA strand, thus resulting in a DSB. The DNA cleavage is performed three nucleotides upstream of the PAM site (Gasiunas *et al.*^2012^). For easier use in genome editing, the crRNA and tracrRNA can be fused tail to head via a linker into a single guiding RNA (gRNA) (Jinek *et al.*^2012^).

This review covers the technical details of the implementation of CRISPR/Cas-mediated genome editing in various yeast species, transcriptional regulation via the enzymatically inactive ‘dead’ dCas9, which binds but does not cut the DNA target (Jinek *et al.*^2012^), and presents examples of applying the technology for engineering of yeast cell factories.

## CRISPR/Cas9 GENOME EDITING IN YEASTS

When Cas9 protein and gRNA are expressed in yeast cells, Cas9 introduces DSBs that must be repaired by the cells via non-homologous end joining (NHEJ) or homologous recombination (HR) (Liu *et al.*^2017^). By supplying a DNA repair template for use in HR, various DNA modifications can be obtained. In the case of efficient cutting, the generated DSBs serve as a negative selection. Thus, there is no need for using a selective marker as in non-CRISPR genome editing methods. Relatively precise and flexible targeting and elimination of the need for positive selection are the two key advantages of the CRISPR/Cas9 technology for yeast genome engineering. The method also allows engineering of diploid and polyploid industrial strains (Ryan *et al.*^2014^; Zhang *et al.*^2014^; Stovicek, Borodina and Forster 2015), which are challenging to manipulate genetically due to the difficulties with modifying multiple alleles and due to the lack of selection markers (Le Borgne 2012). Additionally, by combining several gRNAs, multiple sites can be targeted simultaneously allowing the unprecedented speed of multiple genetic edits (Ryan *et al.*^2014^; Bao *et al.*^2015^; Jakočiūnas *et al.*2015a). On the downside of CRISPR/Cas9, there is a considerable variation in efficiency when targeting different loci, perhaps due to a positional effect of the target region (Smith *et al.*^2016^). At the moment, there also seems to be an upper limit for the number of edits (up to six) that can be introduced simultaneously as every additional introduced DSB decreases the overall yield of surviving clones (Mans *et al.*^2015^; Jakočiūnas *et al.*2015a). Furthermore, CRISPR/Cas9 multiplexing still represents a significant increase in workload for finding correct clones.

Progress has been made on adapting the type II CRISPR/Cas system, described in *Streptococcus pyogenes* (Chylinski *et al.*^2014^), to various yeast species—*Saccharomyces cerevisiae* (Jakočiūnas, Jensen and Keasling 2016), *Yarrowia lipolytica* (Schwartz *et al.*2016a), *Komagataella phaffii* (formerly *Pichia pastoris*) (Weninger *et al.*^2016^), *Kluyveromyces lactis* (Horwitz *et al.*^2015^), *Schizosaccharomyces pombe* (Jacobs *et al.*^2014^), and the pathogenic yeast species *Candida albicans* (Vyas, Barrasa and Fink 2015) and *Cryptococcus neoformans* (Wang *et al.*^2016^). We first discuss the design of the targeting gRNA sequence as a critical aspect of all CRISPR/Cas9 applications. As the vast majority of the studies describing CRISPR/Cas9 genome editing in yeasts have focused on *S. cerevisiae*, the larger section dedicated to this model organism also details some of the more general issues related to the Cas9-mediated genome engineering. For clarity, the studies focusing on the other yeasts are discussed in a separate section.

## COMPUTATIONAL TOOLS FOR gRNA DESIGN IN YEAST

Any ∼20-bp sequence proximal to the PAM site in the genome can serve as the gRNA targeting sequence. The rationale behind careful gRNA selection is to minimize the risk of Cas9-mediated cleavage at unwanted sites in the genome (off-target effects) and maximize the cutting efficiency at the selected site (on-target activity). Other factors may outweigh the best parameters and put additional constraints on the design, e.g*.* position of a target proximal to the beginning of the ORF for generating premature STOP codons or requirement of a target location in promoter/5΄ UTR region in case of gene repression/activation experiments (Mohr *et al.*^2016^). Several web-based tools have been developed to facilitate and automatize the design of gRNA targets (Table 1). Such tools aim mainly at providing guide sequences that minimize the likelihood of off-target effects, matching all possible targets within the given parameters against the reference genome. Some tools provide a list of targets with specified number of mismatches within the entire target sequence or the ‘seed’ sequence (8–12 bp adjacent to the PAM site) (CRISPy (Ronda *et al.*^2014^; Jakočiūnas *et al.*2015a); CRISPRdirect (Naito *et al.*^2015^)), filter out sequences with potential off-target effects (Yeastriction, Mans *et al.*^2015^) or introduce a specificity score based on number of mismatches within the target sequence and rank the targets accordingly (CRISPR-ERA, Liu *et al.*^2015^; Benchling, ATUM gRNA design). CHOPCHOP (Labun *et al.*^2016^) or E-CRISP (Heigwer, Kerr and Boutros 2014) provides the possibility for user-defined parameters of the off-target evaluation. Even though off-target effects are considered unlikely in such a small genome as yeast (Ryan *et al.*^2014^; Jakočiūnas *et al.*2015a), it is advisable to double check the design using yet another tool to avoid introduction of any undesired modifications. Although several potential requirements for gRNA design have been suggested to ensure efficient generation of DSB at the target site, it is still not easy to establish a set of golden rules that would guarantee a success until more experimental data have been acquired. Some of the tools highlight simple features that might influence gRNA efficiency, such as poly T presence in the sequence, GC content (CRISPRdirect) (Naito *et al.*^2015^), AT content or self-complementarity of a gRNA molecule and provide a score based on these parameters (Yeastriction, E-CRISP, CRISPR-ERA) (Heigwer, Kerr and Boutros 2014; Liu *et al.*^2015^; Mans *et al.*^2015^). Other tools such as Benchling have implemented more sophisticated efficiency scores based on an experimental evaluation of a large set of mammalian gRNAs and their sequence features (Doench *et al.*^2014^, ^2016^; Xu *et al.*^2015^). In some cases, users can even choose from several different algorithms of the on-target evaluation (CHOPCHOP, E-CRISP). A few tools also include information on the presence of a specific restriction site in the target sequence (CHOPCHOP, CRISPRdirect, Yeastriction) that might facilitate downstream validation of the cloned target molecule (Mans *et al.*^2015^). CRISPR-ERA or E-CRISP also facilitate designing of a gRNA molecule for engineering applications other than genome editing, e.g. gene repression or gene activation applications. While some of the tools support only one yeast genome, typically *Saccharomyces cerevisiae* reference genome, others provide gRNA design option for several yeast species or various strains of *S. cerevisiae* (Table 1). The CRISPy tool web server implementation, CRISPy-web (Blin *et al.*^2016^), allows for user upload of any GenBank format genome. CRISPRdirect is being frequently updated with new genomes, and CHOPCHOP offers an upload of new genomes on request.

**Table 1.** List of selected web-based bioinformatics tools for gRNA design in yeast.

Name	Link	Reference	Input	Main features	Yeast species
CRISPy	http://staff.biosu stain.dtu.dk/laeb/ crispy_yeast/	Ronda *et al.* (2014 ); Jakočiūnas *et al.* (2015a)	Gene name/ID	Off-target	*S. cerevisiae* reference, CEN.PK
CRISPy-web	http://crispy.second arymetabolites.org	Blin *et al.* (2016)	Gene name/ID, genomic coordinates	Off-target	Any user-submitted genome
CRISPR-ERA	http://crispr- era.stanford.edu/	Liu *et al.* (2015)	Gene name, genomic coordinates, sequence	Off-target, efficiency score, gene repression/activation	*S. cerevisiae* reference
CHOPCHOP v2	http://chopchop.cbu. uib.no	Labun *et al.* (2016)	Gene name, genomic coordinates, sequence	Off-target user defined, on-target algorithm, restriction sites	*S. cerevisiae* reference,
					*C. albicans*,
					*C. tropicalis*,
					*C. glabrata*,
					*P. pastoris*
CRISPRdirect	https://crispr. dbcls.jp/	Naito *et al.* (2015)	Gene name, genomic coordinates, sequence	Off-target, GC content, poly T, restriction sites	*S. cerevisiae*,
					*Sch. pombe*,
					*K. lactis*,
					*Y. lipolytica*,
					*C. albicans*,
					*C. glabrata*
E-CRISPR	http://www.e- crisp.org/	Heigwer, Kerr and Boutros (2014)	Gene symbol, sequence	Off-target, on-target algorithm, gene activation/repression	*S. cerevisiae*,
					*Sch. pombe*
Yeastriction	http://yeastriction. tnw.tudelft.nl	Mans *et al.* (2015)	Gene name	Off-target, AT content, self-complementarity, restriction sites	*S. cerevisiae*, several strains
Benchling	https://benchling. com/ crispr		Gene name, coordinates, sequence	Off-target, on-target algorithm	*S. cerevisiae* reference,
					*Sch. pombe*,
					*C. albicans*,
					*Y. lipolytica*
ATUM gRNA Design Tool	https://www.atum. bio/eCommerce/ cas9/input		Gene name, coordinates, sequence	Off-target	*S. cerevisiae* reference

## CRISPR/Cas9 AND GENOME EDITING IN *SACCHAROMYCES CEREVISIAE*

*Saccharomyces cerevisiae* is an important eukaryotic model organism and also a widely used industrial host for production of fuels, chemicals and recombinant proteins (Borodina and Nielsen 2014; Li and Borodina 2015). Thanks to its excellent HR capability, *S. cerevisiae* is relatively easy to engineer genetically. Below we discuss the ways for delivering Cas9, gRNA and DNA repair templates to *S. cerevisiae* (summarized in Table 2).

**Table 2.** List of available CRISPR/Cas9 tools for yeast.

Reference	Availability	Organism and strain (ploidy)	Cas9 expression (vector, selection marker, promoter)	gRNA expression (vector, selection marker, promoter, terminator)	Application and efficiency
		*S. cerevisiae* genome editing			
DiCarlo *et al.* (2013)	Addgene	*S. cerevisiae* BY4733 (n)	CEN/ARS, *TRP1*, P*_TEF1_*_/GALL_-*Cas9*	2μ, *URA3*, P*_SNR52_*/T*_SUP4_*	Single-gene disruption/marker cassette insertion: 99%
Gao and Zhao (2014)	Addgene	*S. cerevisiae* LPY16936 (n)	2μ, *LEU2*, P_ADH1_-*Cas9*	2μ, *URA3*, P*_ADH1_*-HH ribozyme/HDV ribozyme-T*_ADH1_*	Single-gene disruption: 100%
Ryan *et al.* (2014)	On request	*S. cerevisiae* S288C (n, 2n) ATCC4124 (poly n)	^a^2μ, *kanMX*, P*_RNR2_*_-_*Cas9*^b^	tRNA^Pro^ -HDV ribozyme/T*_SNR52_*	Single/multiple_gene disruption(s): 90%–100%/19%–85%, three-part marker cassette insertion: 70%–85%
Bao *et al.* (2015)	Addgene	*S. cerevisiae* BY4741 (n) CEN.PK2–1c (n)	^a^2μ, truncated *URA3*, P*_TEF1_*-*iCas9*^b^	P*_SNR52_*-crRNA/T*_SUP4_*, P*_RPR1_*-tracrRNA/T*_RPR1_*	Single/multiple-gene disruption: 27%–100%
Zhang *et al.* (2014)	Addgene	*S. cerevisiae* ATCC 4124 (poly n)	CEN/ARS, *natMX*, P*_TEF1_*-*Cas9*	2μ, *hphMX*, P*_SNR52_*/T*_SUP4_*	Single-gene disruption: 15%–60%
Jakočiūnas *et al.* (2015a)	On request	*S. cerevisiae* CEN.PK2–1c (n)	CEN/ARS, *TRP1*, P*_TEF1_*-*Cas9*	2μ, *LEU2*, P*_SNR52_*/T*_SUP4_*	Single/multiple-gene disruption(s): 100%/50%–100%
Mans *et al.* (2015)	Euroscarf	*S. cerevisiae* CEN.PK2–1c (n) CEN.PK113–7D (n) CEN.PK122 (2n)	integr. *can1Δ*::P*_TEF1_*-*Cas9*-*natMX*	2μ, *URA3*, *amdSYM*/*hphMX*/ *kanMX*/*LEU2*/*natMX*/ *HIS3*/ *TRP1*, P*_SNR52_*/T*_SUP4_*	Single-gene deletion: 25%–75%, multiple-gene deletions/multiple-gene cassette insertions: 65%–100%
Stovicek, Borodina and Forster (2015)	Addgene	*S. cerevisiae* CEN.PK113–7D (n) Ethanol Red,	CEN/ARS, *kanMX*, P*_TEF1_*_/ADH1_-*Cas9*	2μ, *natMX*, P*_SNR52_*/T*_SUP4_*	Single-gene disruption and gene cassette insertion: 65%–97%
		CLIB382, CBS7960 (2n)			
Horwitz *et al.* (2015)	On request	*S. cerevisiae* CEN.PK2–1c (n)	integr. gre3Δ::P*_FBA1_*-*Cas9*^c^-*hphMX*	2μ, *URA3*/*HIS3*/*natMX*, P*_SNR52_*/T*_SUP4_*	Single allele swap: 82%–100%, multiple-gene disruptions: 65%–91%, multiple-gene cassette integrations: 4.2%
Tsai *et al.* (2015)	On request	*S. cerevisiae* D452–2 (n)	CEN/ARS, *natMX*, P*_TEF1_*-*Cas9*	2μ, *hphMX*, P*_SNR52_*/T*_SUP4_*	Two part-gene cassettes integration into a single-gene locus: 25%–100%
Laughery *et al.* (2015)	Addgene	*S. cerevisiae* BY4741 (n)	^a^2μ, *LEU2*/*URA3*, P*_TDH3_*-*Cas9*	P*_SNR52_*/T*_SUP4_*	Single-gene disruption: 97%–98%
Lee *et al.* (2015)	Addgene	*S. cerevisiae* S288C (n)	^a^CEN/ARS, *URA3*, P*_PGK1_*-*Cas9*	tRNA^Phe^-HDV ribozyme/T*_SNR52_*	Single/multiple-gene disruption(s): 96%/21%–76%
Jakočiūnas *et al.* (2015b)	On request	*S. cerevisiae* CEN.PK111−27B (n)	CEN/ARS, *TRP1*, P*_TEF1_*-*Cas9*	2μ, *LEU2*, P*_SNR52_*/T*_SUP4_*	Multiple part gene cassette integrations into multiple gene loci: 30%–97%
Ronda *et al.* (2015)	On request	*S. cerevisiae* CEN.PK2–1c (n)	CEN/ARS, *TRP1*, P*_TEF2_*-*Cas9*	2μ, *natMX*, P*_SNR52_*/T*_SUP4_*	Gene cassette integration into multiple intergenic loci: 84–100%
Shi *et al.* (2016)	On request	*S. cerevisiae* HZ848 (n), CEN.PK2–1c (n)	^a^2μ, truncated *URA3*, P*_TEF1_*-*Cas9*	P*_SNR52_*-crRNA/T*_SUP4_*, P*_RPR1_*-tracrRNA/T*_RPR1_*	Long gene fragment integration into multiple genomic loci: 75%–88%
Generoso *et al.* (2016)	Addgene	*S. cerevisiae* CEN.PK113–7D (n) Ethanol Red (2n)	^a^2μ, *kanMX*/*natMX*, P*_ROX3_*-*Cas9*^c^	P*_SNR52_*/T*_SUP4_*	Single and double-gene disruptions: 91%–98%
Jessop-Fabre *et al.* (2016)	Addgene	*S. cerevisiae* CEN.PK113–7D (n) Ethanol Red (2n)	CEN/ARS, *kanMX*, P*_TEF1_*-*Cas9*	2μ, *natMX*, P*_SNR52_*/T*_SUP4_*	Integration of a long gene fragment into a single locus: 95%–100%/multiple loci: 60–70%
Reider Apel *et al.* (2016)	On request	*S. cerevisiae* BY4742 (n)	^a^2μ, *URA3*, *LEU2*, P*_ADH1_*-*Cas9*^c^	tRNA^Tyr^ -HDV ribozyme/T*_SNR52_*	Three part-gene cassette integration into mutiple intergenic loci: 40%–95%
Garst *et al.* (2017)	On request	*S. cerevisiae*	^a^2μ, truncated *URA3*, P*_TEF1_*-*iCas9*^b^	P*_SNR52_*-crRNA/T*_SUP4_*, P*_RPR1_*-tracrRNA/T*_RPR1_*	Single-gene non-sense mutation: 70%–95%
		BY4709 (n)			
		RM11–1 (n)			
Liu *et al.* (2017)	On request	*S. cerevisiae var. boulardii* ATCC MYA-796 (n)	CEN/ARS, *natMX*, P*_TEF1_*-*Cas9*	2μ, *hphMX*, P*_SNR52_*/T*_SUP4_*	Single/double-gene disruption(s):100%/N/A
Nishida *et al.* (2016)	Addgene	*S. cerevisiae*	CEN/ARS, *LEU2*, P*_GAL1_*-*nCas9* (840A)/*nCas9* (D10A)/*n(d)Cas9* (D10A/840A)-*PmCDA1*	2μ, *URA3*, P*_SNR52_*/T*_SUP4_*	Cytidine deaminase-mediated single/double-gene disruption(s): 16%–54%/14%–31%
		BY4741 (n)			
		YPH501 (2n)			
Vanegas, Lehka and Mortensen (2017)	On request	*S. cerevisiae*S288C (n)PJ69–4 (n)	Integr. intergenic X-3::P*_TEF1_*-*Cas9*-*URA3*	CEN/ARS, *LEU2*P*_SNR52_*/T*_SUP4_*	Integration of three-part multiple-gene fragment into an intergenic site: 100%
		*S. cerevisiae* gene activation/repression			
Gilbert *et al.* (2013)	Addgene	*S. cerevisiae*W303	CEN/ARS, *LEU2*, P*_TDH3_*-*dCas9*(-Mxi1)	CEN/ARS, *URA3*, P*_SNR52_*/T*_SUP4_*	Several 10-fold reporter gene transcription repression (CRISPRi)
Farzadfard, Perli and Lu (2013)	Addgene	*S. cerevisiae*W303 (n)	integr. *Δtrp*:: P*_TPGI_*-*dCas9*^c^-VP64-*TRP1*	2μ, *HIS3*/*LEU2*, P*_RPR1_*/T*_RPR1_*	Transcription activation (activator domain)/repression (CRISPRi)
Zalatan *et al.* (2015)	Addgene	*S. cerevisiae*W303 (n)	integr. *Δleu2/*his3::P*_TDH3_*_/_*_GAL10_*-*dCas9*-*CgLEU2*/*HIS3*	CEN/ARS, *URA3*, P*_SNR52_*-gRNA-sc(scaffold)RNA/T*_SUP4_*	Multiple-gene transcription activation (RNA-binding chimeric activators)/repression (CRISPRi)
Chavez *et al.* (2015)	Addgene	*S. cerevisiae*W303 (n)	CEN/ARS, *TRP1*, P*_TDH3_*-*dCas9*-VPR	2μ, *URA3*, P*_SNR52_*/T*_SUP4_*	Transcription activation (multiple activation domains)
Smith *et al.* (2016)	Addgene	*S. cerevisiae*BY4741 (n)	^a^CEN/ARS, *TRP1*/*URA3*, P*_TEF1_*-*dCas9*-Mxi1	P*_RPR1__(TetO)_*/T*_RPR1_*	Transcription repression (repression domain- CRISPRi)
Vanegas, Lehka and Mortensen (2017)	On request	*S. cerevisiaeS288C (n)PJ69–4 (n)*	Integr. Intergenic X-3::P*_TEF1_*-*dCas9*^c^/*dCas9*-VP64-*URA3*	CEN/ARS, *LEU2*P*_SNR52_*/T*_SUP4_*	Transcription activation (activator domain)/repression (CRISPRi)
Deaner and Alper (2017)	On request	*S. cerevisiae*BY4741 (n)	^a^CEN/ARS, *LEU2*, P*_TDH3_*-*dCas9*-Mxi1/P*_TDH3_*-*dCas9*-VPR	P*_SNR52_*/T*_SUP4_*	Graded gene activation/repression (fold transcription activation-gene silencing)
		*K. lactis*			
Horwitz *et al.* (2015)	On request	*K. lactis* ATCC8585 (n)	integr. *Klgal80*::P*_FBA1_*-*Cas9*^c^-*hphMX*	pKD1, *natMX*, P*_SNR52_*/T*_SUP4_*	Multiple-gene cassette insertion into multiple-gene loci: 2.1%
		*Y. lipolytica*			
Schwartz *et al.* (2016a)	Addgene	*Y. lipolytica* ATCC MYA-2613 (n)	^a^CEN, *LEU2*, P_UAS1B8-TEF_-*Cas9*^c^	SCR'-tRNA^Gly^/polyT	Single-gene disruptions (NHEJ/HR): 90%–100%/64%–88% (100% in KU mutant)
Gao *et al.* (2016)	Addgene	*Y. lipolytica* ATCC 201 249 ATCC MYA-2613 (n)	^a^CEN, *LEU2*/*URA3*, P_TEFin_-*Cas9*	P_TEFin_-HH ribozyme/HDV ribozyme-T*_MIG1_*	Single-gene disruption (NHEJ/HR): 62%–98%/72% (94% in KU mutant), multiple-gene disruptions (NHEJ): 19%–37%
Schwartz *et al.* (2016b)	Addgene	*Y. lipolytica* ATCC MYA-2613 (n)	^a^CEN, *LEU2*, P_UAS1B8-TEF_-*Cas9*^c^	SCR'-tRNA^Gly^/polyT	Gene cassette integration into an intergenic locus: 48%–69%
*Ko. phaffii (P. pastoris)*					
Weninger *et al.* (2016)	On request	*Ko. phaffii (P. pastoris)* CBS7435 (n)	PARS1^a^, ZEO, P*_HTX1_*-*Cas9*	P*_HTX1_*-HH ribozyme/HDV ribozyme-T*_AOX1_*	Single-gene disruption: 87%–94%, double-gene disruptions: 69%
*Sch. pombe*					
Jacobs *et al.* (2014)	Addgene	*Sch. pombe* (n)	^a^ars, *ura4*, P*_adh1_*-*Cas9*	P*_rrk1_*/HH ribozyme-T*_rrk1_*	Single-gene disruption (allele swap): 85%–90%
Fernandez and Berro (2016)	On request	*Sch. pombe* FY527 (n) FY528 (n)	^a^ars, *ura4*/*fex1*, P*_adh1_*-Cas9	P*_rrk1_*/HH ribozyme-T*_rrk1_*	Single-gene deletion (ORF removal): 33%
		*C. albicans*			
Vyas, Barrasa and Fink (2015)	On request	*C. albicans* SC5314 (2n)	^a^integr. *ENO1* locus, natMX, P*_ENO1_*-*Cas9*^c^	P*_SNR52_*/T*_ENO1_*	Single/multiple gene disruption(s): 60%–80%/20%
Min *et al.* (2016)	On request	*C. albicans* SC5314 (2n) SN152 (2n)	linear cassette, P*_ENO1_*-*Cas9*^c^	linear cassette, P*_SNR52_*/T*_ENO1_*	Single-gene deletion (ORF replacement with marker cassette): 45%–67%
*C. glabrata*					
Enkler *et al.* (2016)	On request	*C. glabrata* CBS138 (n)	CEN, *TRP1*, P*_CgCYC1_*-*Cas9*	CEN, *LEU2*, P*_CgRNAH1_*/T_CgTY2_	Single-gene disruption
*Cr. neoformans*					
Wang *et al.* (2016)	On request	*Cr. neoformans* serotype D strain JEC21 (n)	^a^linear vector, *URA5*, P*_ACT1_*-*Cas9*	*URA5*, P_CnU6_/polyT	Single-gene disruption (NHEJ/HR): 40%–90%/20%–90%
Arras *et al.* (2016)	On request	*Cr. neoformans* serotype A strain H99 (n)	integr. ‘Safe Heaven’-P*_TEF1_*-*Cas9*	linear vector, P*_ACT1_*-HH ribozyme/HDV ribozyme-T*_TRP1_*	Single-gene deletion (ORF replacement with marker cassette): 65%–70%

The *Cas9* gene is a human codon-optimized version unless otherwise marked. Addgene CRISPR/Cas9 plasmids for use in yeast are available at https://www.addgene.org/crispr/yeast/. Euroscarf deposited vectors can be ordered here www.euroscarf.de.

HH—Hammerhead ribozyme, HDV—hepatitis delta virus ribozyme, iCas9 – mutated ‘hyperactive’ variant, nCas9 – mutated ‘nicking’ variant causing single-strand DNA break, dCas9 – ‘dead’ nuclease activity-lacking variant, PmCDA1 – cytidine deaminase from sea lamprey (*Petromyzon marinus*), Mxi1 – mammalian transcriptional repressor, VP64 – mammalian transcriptional activator domain, VPR—VP64-p65-Rta tripartite activator domain.

a Both components on a single expression element.

b Native *S. pyogenes* Cas9.

c Species codon-optimized Cas9.

### Cas9 expression

The most commonly used *Cas9* gene variant in *S. cerevisiae* has been *Cas9* from *Streptococcus pyogenes*, fused with a nucleolar localization sequence. The DNA sequence of *Cas9* can be either native (Ryan *et al.*^2014^; Bao *et al.*^2015^), human codon-optimized (DiCarlo *et al.*^2013^; Gao and Zhao 2014; Zhang *et al.*^2014^; Laughery *et al.*^2015^; Mans *et al.*^2015^; Stovicek, Borodina and Forster 2015; Jakočiūnas *et al.*2015a) or yeast codon-optimized (Horwitz *et al.*^2015^; Generoso *et al.*^2016^) (Table 2). Only Xu *et al.* (2015) reported the use of *St. thermophilus* CRISPR3 loci-encoded Cas9 (recognizing a different PAM site), albeit with much lower engineering efficiency. The *Cas9* gene was most commonly expressed under the control of constitutive promoters of different strengths from self-replicating low-copy centromeric vectors (DiCarlo *et al.*^2013^; Zhang *et al.*^2014^; Stovicek, Borodina and Forster 2015; Jakočiūnas *et al.*2015a) or high-copy 2μ vectors (Gao and Zhao 2014; Ryan *et al.*^2014^; Bao *et al.*^2015^; Horwitz *et al.*^2015^; Laughery *et al.*^2015^; Generoso *et al.*^2016^) or integrated into the genome (Mans *et al.*^2015^) (Table 2). Expression of *Cas9* on a high-copy vector from a strong constitutive promoter led to a negative influence on the growth of some yeast strains (Ryan *et al.*^2014^; Generoso *et al.*^2016^). However, this problem was not observed in other studies that used the same mode of *Cas9* expression (Gao and Zhao 2014; Bao *et al.*^2015^; Laughery *et al.*^2015^). The toxicity of Cas9 nuclease could be avoided by using weaker promoters for *Cas9* expression (Ryan *et al.*^2014^; Generoso *et al.*^2016^). Overall, the form of *Cas9* expression does not seem to be a critical parameter in CRISPR/Cas9 engineering strategies for *S. cerevisiae*.

### Guide RNA expression

**Figure 1.**
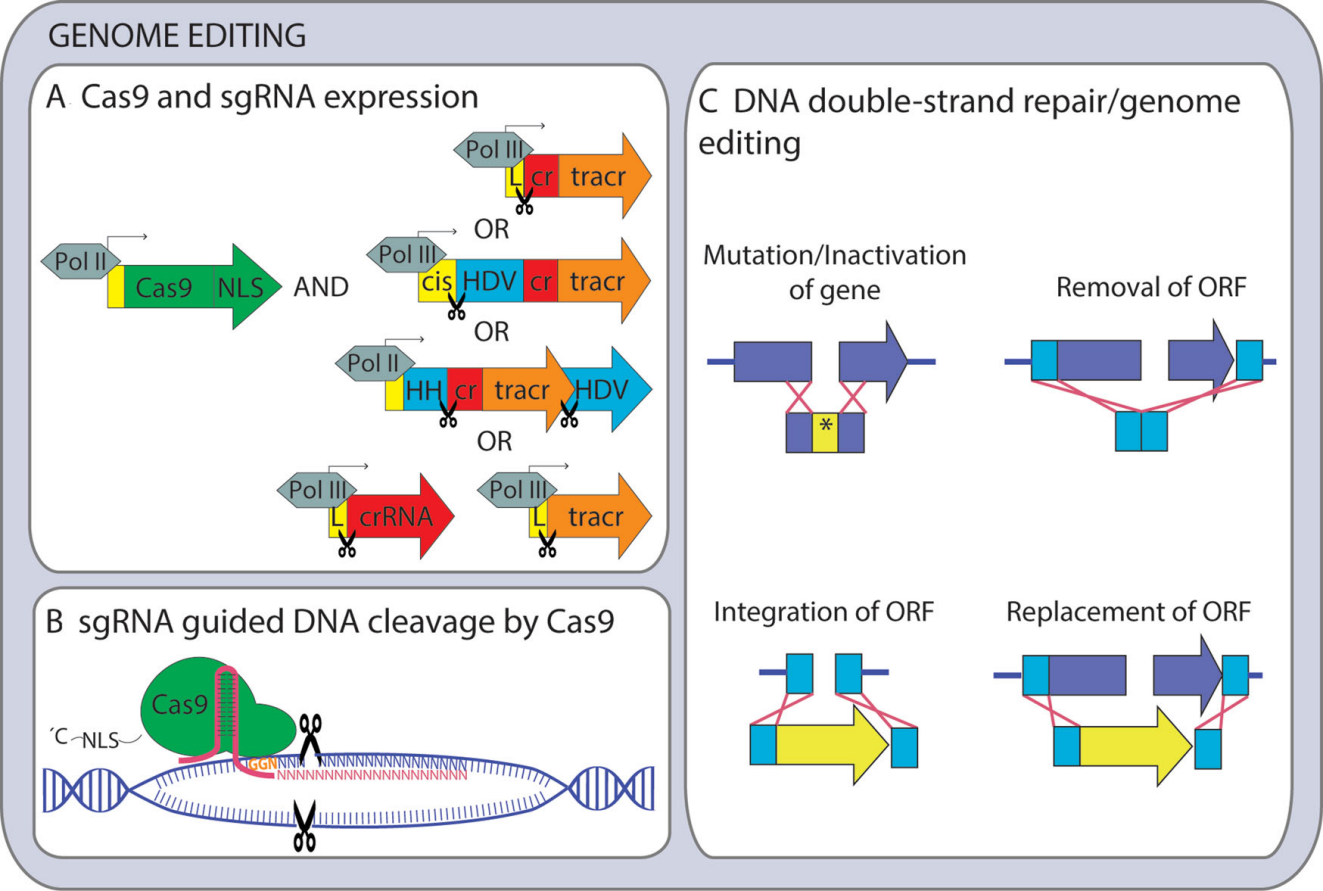
Overview of CRISPR/Cas9-mediated genome editing in yeast. (**A**) Illustration of *Cas9* expression and various means of gRNA expression. (**B**) Mechanism of Cas9/gRNA ribonucleoprotein complex action, NGG (PAM site) highlighted in orange letters. (**C**) Different donor DNA templates for DSB repair. Pol II/III—RNA Polymerase II/III, NLS—nucleolar localization sequence, cis—cis regulatory element (tRNA), L—self-cleaved leader sequence (*SNR52*), cr—crRNA, tracr—tracrRNA, HH—hammerhead ribozyme, HDV—hepatitis delta virus ribozyme, *—STOP codon.

Design, expression and delivery of the gRNA components are crucial parameters for successful CRISPR/Cas9 engineering. In *S. cerevisiae*, the most common strategy has been to express a chimeric gRNA molecule from a high-copy vector to ensure its abundant expression (Table 2). Both ends of the gRNA molecule must be precisely defined to create a functional Cas9/gRNA complex. Functional gRNA transcription has been achieved using (i) an RNA polymerase III (Pol III) promoter that provides a transcript with a leader sequence cleaved during molecule maturation (DiCarlo *et al.*^2013^; Farzadfard, Perli and Lu 2013); (ii) Pol III promoters containing *cis*-regulatory elements within the mature RNA molecule (tRNA) combined with a ribozyme, cleaving the transcript on its 5΄ end (Ryan *et al.*^2014^); and (iii) an RNA polymerase II (Pol II) promoter, if the gRNA molecule is flanked with two ribozymes executing cleavage on both ends of the molecule (Gao and Zhao 2014) (Fig. 1). Besides the chimeric gRNA approach, separate expression of a targeting crRNA array driven by a Pol III promoter, processed by native RNA processing enzymes, and tracrRNA transcribed from another Pol III promoter has been reported (Bao *et al.*^2015^). The expression cassette containing *SNR52* promoter and *SUP4* terminator, an approach shown to produce prokaryotic tRNA molecules in yeast (Wang and Wang 2008), was successfully used for targeting a single gene in haploid or diploid laboratory strains with engineering efficiencies reaching 100% (DiCarlo *et al.*^2013^; Horwitz *et al.*^2015^; Laughery *et al.*^2015^; Mans *et al*. 2015; Jakočiūnas *et al.*2015a; Generoso *et al.*^2016^) (Table 2). It is important to mention that engineering efficiencies discussed in this review are defined as the number of clones with the desired genomic edit per number of clones surviving after the transformation. Such values should not be mistaken with transformation efficiency values used traditionally in non-CRISPR engineering studies as these relate to the number of viable cells in the transformation reaction (Storici *et al.*^2003^; Alexander, Doering and Hittinger 2014). Although some studies also provide transformation efficiency values that reflect the number of cells not surviving the transformation (DiCarlo *et al.*^2013^; Stovicek, Borodina and Forster 2015), many others do not, leaving the engineering efficiency as the only relevant benchmark. The *SNR52* promoter/*SUP4* terminator setup also allowed for gene deletion in various diploid industrial strains with efficiencies ranging between 65% and 78% (Stovicek, Borodina and Forster 2015) and even polyploid strains with 15%–60% efficiency (Zhang *et al.*^2014^) (Table 2). The expression of a gRNA fused to a *Hepatitis delta* virus (HDV) ribozyme controlled by a tRNA promoter and *SNR52* terminator led to almost 100% gene deletion efficiency in a diploid laboratory strain and more than 90% in a polyploid industrial strain (Ryan *et al.*^2014^). As for the third mentioned approach, a gRNA molecule flanked with Hammerhead (HH) and HDV ribozymes on the 5΄ and 3΄ end, respectively, expressed from *ADH1* promoter also enabled efficient gene disruption in a laboratory strain (Gao and Zhao 2014). The crRNA array method achieved efficiencies of 76%–100% in a laboratory strain after several days of outgrowth of the transformed cells (Bao *et al.*^2015^).

**Figure 2.**
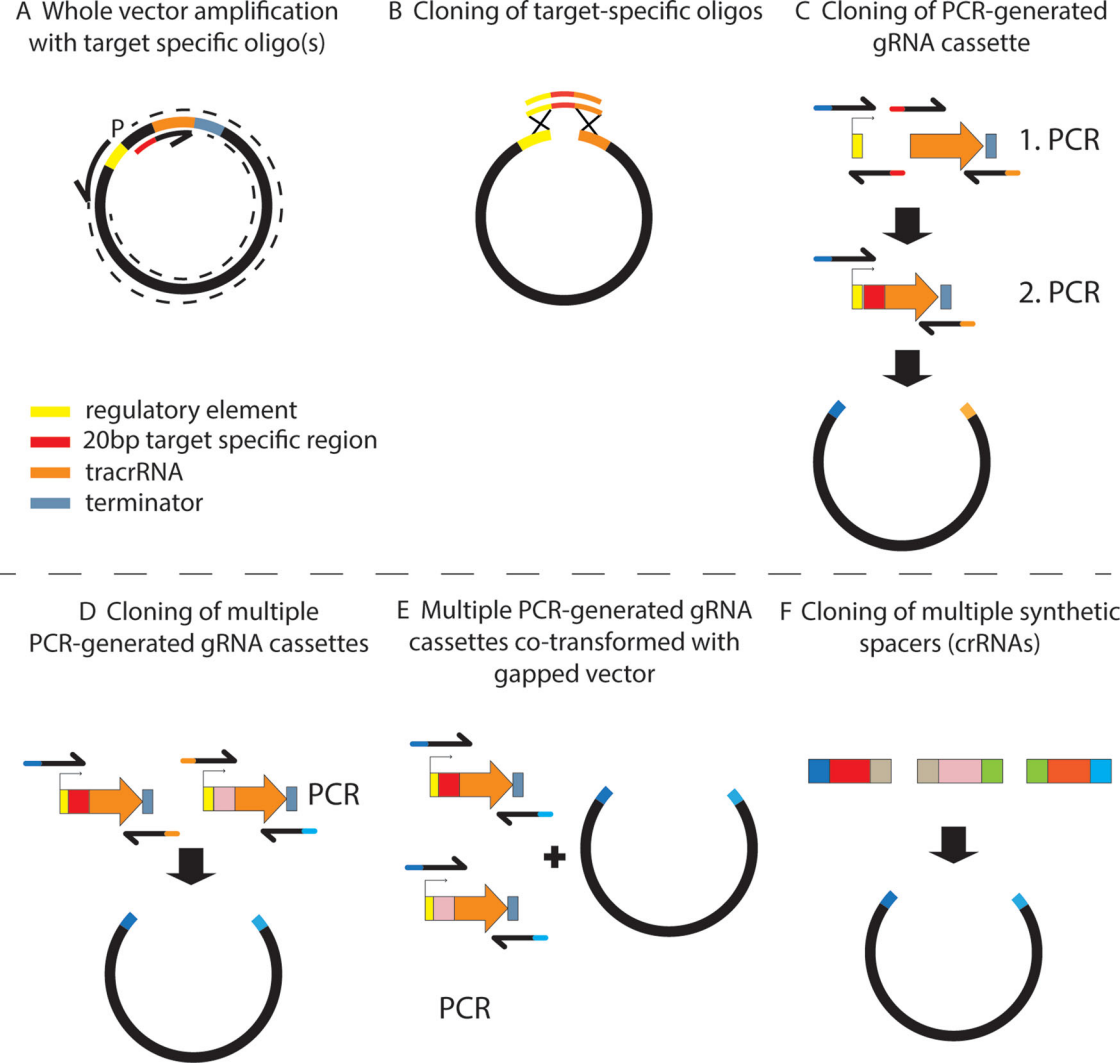
Generation of specific gRNA expression cassettes. (**A**) Vector can be circularized via ligation (one oligo phosphorylated) (Jakočiūnas *et al.*2015a; Stovicek, Borodina and Forster 2015), ligation-free primer extension reaction (Tsai *et al.*^2015^; Ryan, Poddar and Cate 2016), Gibson assembly or recombination *in vivo* (pair of oligos overlapping at the specific gRNA target sequence) (Generoso *et al.*^2016^). (**B**) Short synthetic oligos are cloned via e.g. Gibson assembly (oligos with overhangs homologous to the ends of the digested vector) (Reider Apel *et al.*^2016^), restriction cloning (oligos with overhangs complementary to a particular restriction site) (Laughery *et al.*^2015^), modular cloning (seamless assembly using type IIS restriction enzymes, oligos with overhangs complementary to a particular restriction site) (Lee *et al.*^2015^; Vyas, Barrasa and Fink 2015) or *in vivo* in yeast (Mans *et al.*^2015^). (**C**) Cloning of the two-step PCR generated gRNA cassette via Gibson assembly (DiCarlo *et al.*^2013^) or restriction cloning (Chin *et al.*^2016^). (**D**) Several single gRNA cassettes cloned via Gibson assembly (Weninger *et al.*^2016^), restriction cloning (Ryan *et al.*^2014^) or modular assembly (Lee *et al.*^2015^). Alternatively, two-gRNA cassette fragments in opposite orientation can be amplified in one reaction and cloned (Mans *et al.*^2015^; Generoso *et al.*^2016^). (**E**) Pool of several single gRNA cassettes transformed to yeast cells with a gapped vector for *in vivo* recombination (Horwitz *et al.*^2015^). (**F**) crRNA array is cloned via Golden gate assembly of short synthetic fragments with homologous overlaps (Bao *et al.*^2015^).

When a researcher decides to engineer a targeted genomic locus, only the ∼20 bp recognition sequence of a gRNA molecule needs to be modified to redirect the Cas9/gRNA complex to a particular target site. Several ways of obtaining an expression vector with a customized gRNA molecule have been described (Fig. 2). Several studies exchanged the recognition sequence of a gRNA vector using whole vector amplification with primers containing a new target-specific 20-bp region. Vector circularization was achieved via PCR with a phosphorylated primer, followed by ligation (Stovicek, Borodina and Forster 2015; Jakočiūnas *et al.*2015a), *in vivo* in yeast or *in vitro* Gibson assembly using two oligos overlapping at the target sequence (Generoso *et al.*^2016^), or via restriction-free cloning (van den Ent and Löwe 2006) with two 60-bp complementary oligos containing a target sequence (Ryan and Cate 2014). In other studies, two target-specific complementary oligos containing sequences overlapping with the gRNA cassette were cloned into a vector using Gibson assembly (Reider Apel *et al.*^2016^) or restriction sites located between promoter and the gRNA structural part (Laughery *et al.*^2015^; Lee *et al.*^2015^), or transformed directly into yeast along with the digested expression vector (Mans *et al.*^2015^). Alternatively, the gRNA cassette was amplified using two-step fusion PCR and cloned via Gibson assembly (DiCarlo *et al.*^2013^) and standard restriction cloning (Chin *et al.*^2016^) or transformed along with a digested expression vector for *in vivo* vector gap repair in yeast (Horwitz *et al.*^2015^). To omit the PCR amplification step, customized gRNA cassettes can be synthesized as gene blocks and integrated into a vector via restriction cloning (Zhang *et al.*^2014^) or USER assembly (Ronda *et al.*^2015^; Jakočiūnas *et al.*2015b). Lastly, Golden gate cloning of synthetic parts of the crRNA array has also been shown (Bao *et al.*^2015^).

In summary, researchers can choose from a number of cloning systems for generation of a target gRNA molecule and can also benefit from online tools facilitating the particular cloning design (Laughery *et al.*^2015^; Mans *et al.*^2015^) or detailed (Ryan, Poddar and Cate 2016) and straightforward protocols (Jakočiūnas *et al.*2015a). However, even in its simplest version, the CRISPR/Cas9 engineering relies on a gRNA vector construction, which can be laborious and costly. The gap repair approach developed by Horwitz *et al.* (2015) skips the cloning step. However, it requires longer DSB repair templates, high efficiency of HR in the strain and may result in a non-equimolar expression of the gRNAs when multiplexing. A lower efficiency of engineering with vectors based on *in vivo* assembly has been documented (Mans *et al.*^2015^; Generoso *et al.*^2016^).

One can also choose to express Cas9 and gRNA from a single vector (Ryan *et al.*^2014^; Bao *et al.*^2015^; Laughery *et al.*^2015^; Generoso *et al.*^2016^). However, due to the large size of the *Cas9* gene, generation of a gRNA via the whole plasmid PCR amplification (Ryan and Cate 2014) might be difficult. Such a system is also not compatible with the gap repair gRNA generation as this one requires expression of Cas9 prior the transformation with the gRNA vector (Walter, Chandran and Horwitz 2016).

### Multiplexing gRNA expression

In *S. cerevisiae*, efficient HR system allows creating multiple genomic changes simultaneously using CRISPR/Cas9. For each genome edit, an individual gRNA must be expressed and a repair template delivered into the cells. The multiple gRNA expression has been achieved using (i) several vectors with different selection markers containing up to two different gRNA expression cassettes (Mans *et al.*^2015^), (ii) a single expression vector carrying several gRNA cassettes (Ryan *et al.*^2014^; Lee *et al.*^2015^; Jakočiūnas *et al.*2015a), (iii) an array of different interspaced crRNAs (Bao *et al.*^2015^) or (iv) different linear gRNA expression cassettes transformed along with a single gapped expression vector (Horwitz *et al.*^2015^). When up to three different vectors, each carrying two gRNA expression cassettes were transformed, 100%, 70% and 65% efficiency of two, four or six gene deletions was achieved, respectively (Mans *et al.*^2015^). The expression of five individual gRNAs from one vector provided target efficiencies ranging between 50% and 100% (Jakočiūnas *et al.*2015a). Ryan *et al.* (2014) reported successful gene deletion of two or three genes with efficiencies of 86% and 81% in haploid and 43% and 19% in diploid strains using HDV-gRNA expression cassettes in a single expression vector, respectively. Cloning of crRNA arrays targeting three different genes achieved engineering efficiencies ranging between 27% and 100% (Bao *et al.*^2015^). The gap repair approach using the transformation of three different gRNA cassettes and a single open vector enabled recovery of 64% positive three-gene deletion mutants (Horwitz *et al.*^2015^) (Table 2).

As described above, all setups enabled successful marker-free multiplexed genome editing in *S. cerevisiae*. However, despite the reported encouraging results, yeast strains can differ in engineering efficiencies given rather by their nature than differences in the described procedures. Diploid or polyploid industrial strains can be especially difficult to engineer (Zhang *et al.*^2014^; Stovicek, Borodina and Forster 2015; Generoso *et al.*^2016^), and multiplexing can create more work on the other end to identify the correct clones. The CRISPR/Cas9 system also greatly facilitates the sequential introduction of multiple genomic edits. For repeated rounds of editing, the strain is cultivated in the absence of selection pressure for gRNA vector, while maintaining selection pressure for Cas9 vector. Then a new gRNA vector can be introduced to accomplish the next round of genetic modifications. In the final strain, both vectors can be removed in the absence of selection pressure to generate a strain free of selection markers (Stovicek, Borodina and Forster 2015; Jessop-Fabre *et al.*^2016^).

### DNA repair templates

As mentioned above, the dominant mode of DSB repair in *S. cerevisiae* is HR when a homologous donor template is available. NHEJ response in *S. cerevisiae* provides unpredictable results at target sites and severely decreases overall yield of surviving cells (DiCarlo *et al.*^2013^; Mans *et al.*^2015^; Stovicek, Borodina and Forster 2015). It has been shown that short single-strand (Generoso *et al.*^2016^) or double-strand DNA donor oligos (DiCarlo *et al.*^2013^) sharing homology with a target site can serve as the simplest repair template. The donor oligo can be of various lengths, ranging between 80 and 120 bp, and can introduce various changes such as a premature STOP codon (DiCarlo *et al.*^2013^), a heterologous disrupting sequence (Horwitz *et al.*^2015^), a barcode (Ryan *et al.*^2014^) for easier genotyping or an entire ORF deletion (Mans *et al.*^2015^) (Fig. 1). The PAM site should always be removed from the donor sequence to prevent the cutting by Cas9 (DiCarlo *et al.*^2013^). The repair template can also be delivered as a part of an expression vector (Bao *et al.*^2015^; Garst *et al.*^2017^). Longer gene expression cassettes with at least 40-bp homology to the target site can also be used as repair templates. They enable integration of larger DNA fragments, e.g. carrying gene expression cassettes (DiCarlo *et al.*^2013^; Stovicek, Borodina and Forster 2015). The CRISPR/Cas9 approach has also been combined with *in vivo* assembly of several overlapping DNA parts (Fig. 1). Ryan *et al.* (2014) reported 70%–85% efficiency for assembly of a gene expression cassette consisting of three parts with 50 bp overlaps to a targeted locus in a diploid or polyploid strain. In another study, one transformation event enabled integration of six overlapping gene expression cassettes into a single-gene locus while another gene was deleted using a short oligo simultaneously (Mans *et al.*^2015^). A multiplex approach CasEMBLR demonstrated assembly of five overlapping DNA parts per locus in up to three different loci simultaneously with an efficiency ranging between 30% and 97% (Jakočiūnas *et al.*2015b). A metabolic pathway consisting of 11 genes on six DNA parts flanked with 500 bp arms homologous to three independent loci was used as a DSB repair template in the gap-repair gRNA delivery approach resulting in 4% efficiency of the pathway assembly (Horwitz *et al.*^2015^). Tsai *et al.* (2015) integrated two copies of a multigene pathway consisting of six genes on four DNA parts with 300 bp homologous arms into two different gene loci with 25%–100% efficiency. As the integration of a gene expression cassette into an ORF may influence expression of the heterologous gene (Stovicek, Borodina and Forster 2015), several toolkits targeting intergenic regions providing reliable level of gene expression have been developed. Three-DNA part gene expression cassettes with 1 kbp homologous arms used as a donor template resulted in 40%–95% integration efficiency depending on the particular site targeted (Reider Apel *et al.*^2016^). Although versatile, *in vivo* CRISPR/Cas9-mediated assembly requires tedious multiplex genotyping. Thus, preassembly of donor templates with sufficiently long homologous arms might be an alternative option to omit this. Using the system developed by Bao *et al.* (2015), preassembled large metabolic pathways were integrated into transposable Ty elements in multiple copies with efficiencies more than 80% (Shi *et al.*^2016^). Ronda *et al.* (2015) targeted multiple validated intergenic loci with preassembled gene expression cassettes reaching efficiencies of 84% with three simultaneous integrations. When using the marker-free variant of previously designed integrative vectors (Jensen *et al.*^2014^; Stovicek *et al.*^2015^) targeting intergenic loci, integration of up to six heterologous genes was achieved with 70% efficiency (Jessop-Fabre *et al.*^2016^). In summary, due to the high efficiency of HR, large pathways can be assembled directly *in vivo* omitting *in vitro* cloning steps. However, the preassembly of donor DNA fragments always leads to higher integration efficiencies and does not require subsequent extensive genotyping.

Taken together, mainly linear DNA fragments of different length have been successfully used for efficient DSB repair. However, a recent study demonstrated that episomal vectors that contain both a gRNA expression cassette and a DNA repair template could also be used in the yeast *S. cerevisiae* (Garst *et al*. 2017). In a proof-of-concept experiment, *ADE2* gene was mutated with 95% efficiency in a laboratory strain and with 70% efficiency in a wine strain. A particular advantage of this method is that the combined DNA elements, which contain a gRNA and a corresponding repair template, are small enough (∼200 bp) to be synthesized by high-throughput oligomer synthesis on arrays. Combined with a high transformation efficiency of episomal vectors into yeast, this enables generation of large strain libraries.

### Direct DNA editing using CRISPR-cytidine deaminase fusion

A method for CRISPR-based targeted DNA mutagenesis was described by taking advantage of an activation-induced cytidine deaminase (AID), which is normally responsible for somatic hypermutation of the variable regions of antibodies (Nishida *et al.*^2016^). When AID was expressed as a fusion with dCas9 in *S. cerevisiae*, AID deaminated deoxycytidine to deoxyuridine 15–19 bases upstream of the PAM sequence on the non-complementary strand to gRNA, effectively creating C→G/T point mutations. The efficiency of gene inactivation using this approach was 16%–47%, depending on the chosen target site. The advantage of the CRISPR-AID method is a reduced toxicity in comparison to the nuclease-based CRISPR approaches (Nishida *et al.*^2016^).

## CRISPR/Cas9 GENOME EDITING IN DIFFERENT YEAST SPECIES

### Kluyveromyces lactis

*Kluyveromyces lactis* is used industrially for the production of recombinant proteins, fermented dairy products and some metabolites (Spohner *et al.*^2016^). Horwitz *et al.* (2015) demonstrated CRISPR/Cas9 genome editing in an industrial strain of *K. lactis*. The 2μ element in the expression vector was exchanged for the pKD1 vector-stabilizing element. To decrease the NHEJ activity in *K. lactis*, the authors deleted *YKU80* gene. Although with low efficiency (2.3%), the method allowed integration of three six-gene DNA parts into three individual chromosomal loci (Horwitz *et al.*^2015^).

### Yarrowia lipolytica

*Yarrowia lipolytica* is the most studied oleaginous yeast and is applied in the biotechnology industry for the production of lipase, citric acid, lactone fragrances and recently also ω-3 fatty acids (Thevenieau, Nicaud and Gaillardin 2009; Xue *et al.*^2013^). Several recent studies have demonstrated the potential of the CRISPR/Cas9 system in this yeast. Schwartz *et al.* (2016a) constructed *Yarrowia* codon-optimized *Cas9* and hybrid SCR1΄-tRNA promoter for gRNA expression on a centromeric vector (Schwartz *et al.*2016a). It enabled efficient NHEJ-generated gene deletions. More than 50% or 90% of the cells acquired a gene deletion after 2 or 4-day outgrowth of the transformed cells, respectively. HR-mediated gene deletions with a donor fragment with 1-kbp homologous arms were also obtained with high efficiency. The HR-mediated repair was pronounced in KU70 mutant, lacking NHEJ-mediated response (Schwartz *et al.*2016a). A possibility of multiplex gene deletion in *Y. lipolytica* was also demonstrated (Gao *et al.*^2016^). Here a vector was designed to carry *Yarrowia* codon-optimized *Cas9* gene driven by the strong, endogenous *TEF1* promoter, and also gRNAs flanked with the HH and HDV ribozymes expressed from the *TEF1* promoter. In the absence of donor DNA, NHEJ-mediated gene nonsense mutations occurred with efficiencies of 85%, 36% or 19% for one, two or three targeted genes, respectively, after 4 days of outgrowth of the transformed cells. Furthermore, HR-mediated gene disruption was shown when the donor template was delivered on the Cas9/gRNA vector, with higher rates in KU70/80 mutants (Gao *et al.*^2016^). CRISPR/Cas9 also allowed the development of a toolkit for integration of donor cassettes which were delivered into the cells by a separate replicative vector requiring an additional selection during the transformation (Schwartz *et al.*2016b). In an NHEJ-positive strain, 5 out of 17 tested locations were targeted with integration efficiencies from 48% to 69%, while 3 sites showed <6% and the remaining 9 sites did not show any positive integration. Sequential markerless integration of a metabolic pathway into the described loci was shown (Schwartz *et al.*2016b).

### Komagataella phaffii (formerly Pichia pastoris)

*Komagataella phaffii* (*P. pastoris*) is an important recombinant protein producer due to its excellent folding and secretion capability. However, it is poor in HR, which makes it very hard to engineer. Weninger *et al.* (2016) extensively tested different modes of expression of the *Cas9* gene and gRNA molecules. Use of a low-copy ARS element vector with bidirectional native *HXT1* promoter driving the expression of human codon-optimized *Cas9* and HH/HDV-ribozyme-flanked gRNA transcript resulted in up to 90% of single-gene nonsense mutations. When two genes were targeted, nonsense mutations in both ORFs were observed with a frequency of 69%. Although a donor template with 1-kbp homologous arms was provided, only very low integration efficiency (2%) occurred suggesting that NHEJ remained the dominant way of DSB repair (Weninger *et al.*^2016^).

### Schizosaccharomyces pombe

The fission yeast *Sch. pombe* is an important model organism for the study of eukaryotic cellular biology and in particularly cell cycle regulation (Hoffman, Wood and Fantes 2015). Jacobs *et al.* (2014) used *rrk1* promoter for expression of gRNA molecule as it provides a defined 5΄-leader, cleaved during maturation. The 3΄-end of the gRNA molecule was fused to the HH ribozyme, as *rrk1* is a Pol II promoter resulting in polyadenylation of mature RNAs. Expression of gRNA and Cas9 separately on two low-copy ARS-containing vectors (or together on one vector to minimize the observed negative influence of Cas9 expression on cell growth) led to the 85%–98% efficiency of the target modification when a PCR-amplified mutated allele was used as donor template (Jacobs *et al.*^2014^). A similar system enabled construction of a single-gene deletion with 33% efficiency (Fernandez and Berro 2016).

### Pathogenic yeasts

Targeted gene deletions are necessary for the study of gene functions in virulence models. In the most prevalent yeast pathogen—*Candida albicans*—the absence of haploid state and frequent aneuploidy of clinical isolates makes gene deletions very tedious. In the absence of autonomously replicating vectors, CRISPR/Cas9 was implemented via integrating *Cas9* controlled by *ENO1* promoter and gRNA expressed from *SNR52* promoter into *C. albicans* genome (Vyas, Barrasa and Fink 2015). The *Cas9* gene was codon-optimized for CTG clade yeasts. In ‘solo’ approach, gRNA expression cassette was integrated into a strain already expressing Cas9. In the ‘duet’ approach, both expression cassettes were integrated in a single transformation. Both ‘solo’ and ‘duet’ systems resulted in an acceptable gene deletion efficiency of 60%–80% and 20%–40%, respectively. The more efficient ‘solo’ system was then used for generation of deletions in several genes or deletion of two homologous genes with a single targeting gRNA molecule. Moreover, successful nonsense mutations in three different loci combining the solo and duet system for delivery of two different gRNA cassettes were documented (Vyas, Barrasa and Fink 2015). A possibility of transient expression of linear cassettes carrying both components was shown. A single gene was replaced with a linear marker gene cassette reaching more than 50% efficiency, while the linear gRNA and *Cas9* cassettes were lost at the same time (Min *et al.*^2016^).

Another pathogenic yeast *Cryptococcus neoformans* exhibits a low rate of HR that hampers its manipulation and thus functional gene analysis. Two studies have demonstrated the CRISPR/Cas9 system capacity to generate nonsense mutations and to stimulate HR response in different serotypes of *Cr. neoformans* (Arras *et al.*^2016^; Wang *et al.*^2016^). As circular molecules are not stable in *Cr. neoformans*, linear DNA vectors were used for expression of Cas9 nuclease and gRNAs. gRNAs were expressed from CnU6 promoter and terminated by 6T terminator (Wang *et al.*^2016^). Alternatively, the *Cas9* gene was integrated into the genome and a linear vector was used for expression of a gRNA molecule flanked with HH and HDV ribozymes from a Pol II promoter (Arras *et al.*^2016^). The introduction of nonsense mutations was achieved without donor DNA with efficiency above 80%. Mutated allele used as a donor template resulted in HR-mediated allele exchange when selecting for a particular phenotype. Full removal of an ORF occurred with frequencies of 20%–90% when a donor marker gene was fused to the Cas9/gRNA cassette followed by spontaneous loss of the Cas9/gRNA part eliminating thus the persistence of the CRISPR/Cas9 system (Wang *et al.*^2016^). Gene deletions were obtained in different serotypes of *Cr. neoformans* by using a marker cassette with homologous arms to the given ORF. Stimulation of HR led to 70% success rate for obtaining the mutants (Arras *et al.*^2016^).

Another pathogenic yeast with a dominant NHEJ pathway, *C. glabrata* was demonstrated to be amenable to the CRISPR/Cas9-mediated engineering (Enkler *et al.*^2016^). Here two centromeric vectors carrying *Cas9* and gRNA expression cassette were used. Although adoption of *Saccharomyces cerevisiae* system (DiCarlo *et al.*^2013^) for expression of a gRNA appeared to be feasible, specific *C. glabrata* adjustments (RNAH promoter, tRNA terminator) led to better performance of the system (Enkler *et al.*^2016^). Besides efficient generation of indels by NHEJ, deletion of a reporter gene using a donor marker cassette with relatively short homologous arms was achieved with increased HR rates (Enkler *et al.*^2016^).

## TRANSCRIPTIONAL REGULATION VIA CRISPR

Targeted regulation of gene expression is important both in the context of metabolic engineering and functional genomics. The CRISPR method has been adapted both for activation and repression of gene transcription in *Saccharomyces cerevisiae*, but so far not in other yeast species.

**Figure 3.**
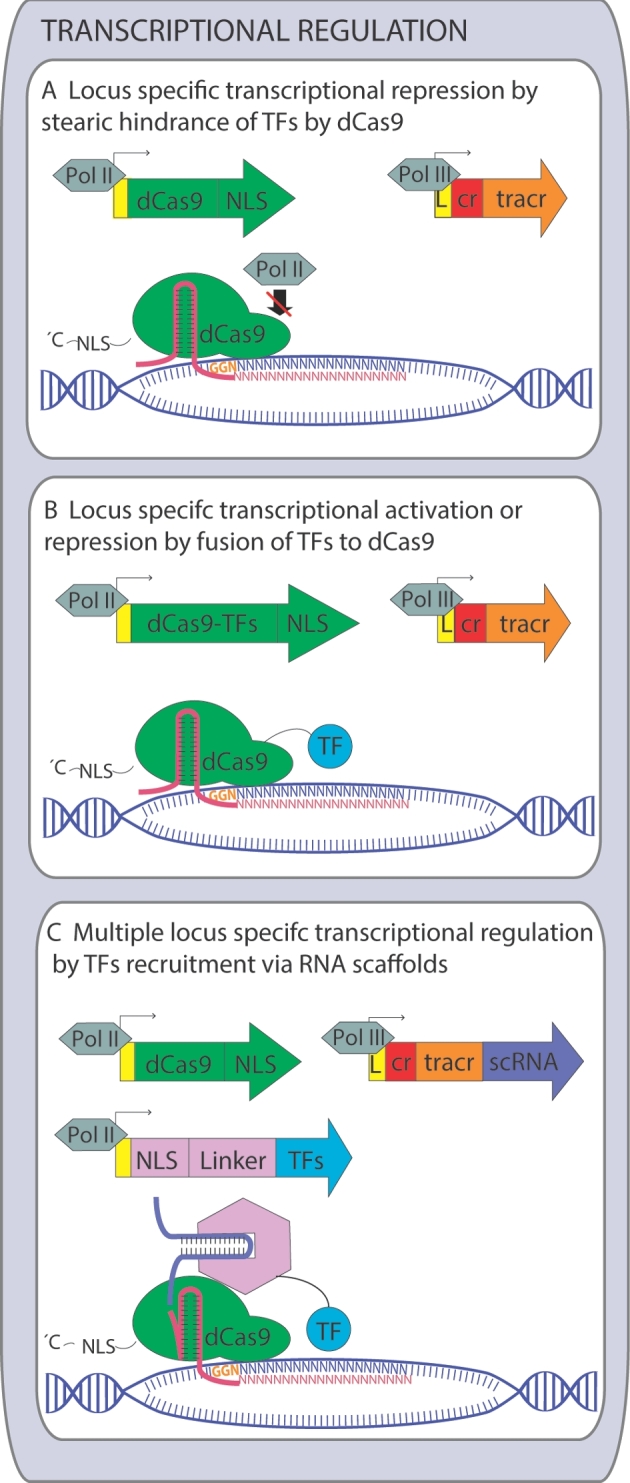
Overview of transcriptional control via CRISPR/Cas9 in yeast. (**A**) Steric block of transcriptional initiation/elongation by catalytically inactive (‘dead’) dCas9 bound in the promoter region. (**B**) Transcriptional activation/repression using dCas9 fused to transcriptional activator/repressor domains. (**C**) Multiple transcriptional regulation action using effector proteins recruited by RNA scaffolds. Pol III—RNA Polymerase III, NLS—nucleolar localization sequence, L—self-cleaved leader sequence (*e.g.* SNR52), cr—crRNA, tracr—tracrRNA, TF—transcription factor, scRNA—scaffold RNA, Linker—scaffold RNA-binding linker protein domain.

Qi *et al.* (2013) generated an enzymatically inactive variant of Cas9 by mutation of both nuclease sites (D10A and H840A) and showed that this null-nuclease dCas9 when targeted to a coding region of a gene caused transcriptional repression in *Escherichia coli*. In this approach, termed CRISPR interference (CRISPRi), dCas9 sterically blocks the binding and action of RNA polymerase. In a follow-up study, dCas9 was guided to a promoter region, resulting in efficient gene repression in *S. cerevisiae* (Gilbert *et al.*^2013^). The repression could be further enhanced by fusing a repressor domain to dCas9 (Fig. 3). GFP fluorescence was reduced 18-fold when the *TEF1* promoter driving the GFP expression was targeted by dCas9, and the fluorescence decreased 53-fold when the same region was targeted by dCas9 fused to a mammalian transcriptional repressor domain Mxi1 (Gilbert *et al.*^2013^).

Farzadfard, Perli and Lu (2013) fused dCas9 to an activator domain (VP64) instead. The resulting chimeric protein could both repress and activate gene expression depending on the targeting site in the promoter region. When dCas9-VP64 was targeted to the region upstream the TATA box of the minimal *CYC1m* promoter, the promoter was activated. Targeting the sites immediately adjacent to the TATA box or transcriptional start site repressed the expression from P*_CYC1_*_m_. The obtained activation level was not very high, max 2.5-fold. To achieve a higher level of activation, the authors created a synthetic promoter by arraying multiple operators upstream the P*_CYC1_*_m_. The activation level increased proportionally to the number of operators, reaching 70-fold activation for 12 operators (Farzadfard, Perli and Lu 2013).

Fusion of dCas9 to a tripartite activator (VPR) composed of three strong activation domains (VP64, p65 and Rta) resulted in 38 and 78-fold activation of promoters P*_HED1_* and P*_GAL7_*, respectively. Fusion of dCas9 with VP64 only gave 9 and 14-fold activation of the same promoters (Chavez *et al.*^2015^).

Zalatan *et al.* (2015) undertook a different approach to achieve targeted upregulation and downregulation. Instead of fusing activation or repression domains to dCas9, they included effector protein recruitment domains into the guide RNA (Fig. 3). In the same strain, they expressed dCas9 and regulation proteins, fused with RNA-binding domains. They termed the resulting gRNAs with protein recruitment capabilities ‘scaffold RNA’ (scRNA). Gene activation using scRNA binding VP64 activation domain was 20 to 50-fold, much higher than the activation achieved with dCas9-VP64 fusion. Several hairpins could be combined in a single scRNA, which allowed amplification of activation or combination of repression and activation of different sites (Zalatan *et al.*^2015^).

In addition to the studies mentioned above focusing on the on/off states of gene expression, grade modulation of gene expression using dCas9 fused to either an activation or repression domain was shown (Deaner and Alper 2017). This was achieved by changing the gRNA target location and thus recruiting the dCas9-activator/repressor complex to different positions in gene promoters. It resulted in a dynamic range of gene expression from almost silenced gene to its several 10-fold overexpression related to the proximity of the dCas9-based regulators to the core of the promoter. The graded gene expression enabled tuning of metabolic pathways and optimization of the desired phenotypes in several metabolic engineering applications (Deaner and Alper 2017).

## APPLICATION OF CRISPR/Cas9 FOR ENGINEERING OF YEAST CELL FACTORIES

**Figure 4.**
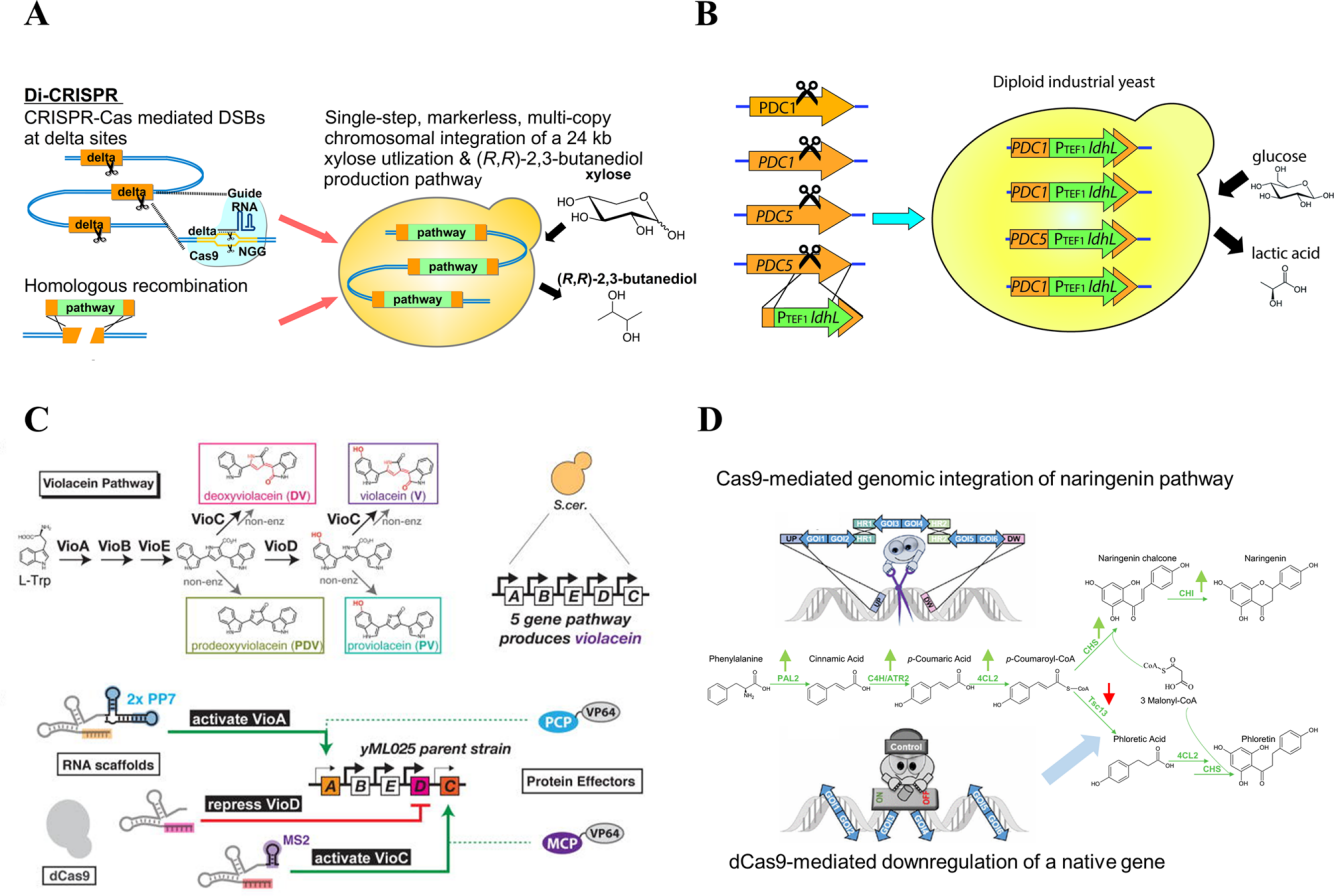
Application of CRISPR/Cas9 systems for engineering of yeast cell factories. (**A**) Production of (R,R)-2,3-butanediol from xylose. Multicopy one-step integration of the xylose utilization and (R,R)-2,3-butanediol pathways into Ty-element delta sites in the genome (The figure is reprinted with permission from Elsevier: Shi *et al.* A highly efficient single-step, markerless strategy for multicopy chromosomal integration of large biochemical pathways in *Saccharomyces cerevisiae*. *Metab Eng* 2016;**33**:19–27.). (**B**) Production of lactic acid from glucose in an industrial yeast strain, one-step disruption of two genes in diploid strain and simultaneous integration of lactate dehydrogenase genes from *L. plantarum* (*ldhL*) (Stovicek, Borodina and Forster 2015). (**C**) Production of deoxyviolacein, violacein, prodeoxyviolacein and proviolacein from glucose. Transcriptional regulation (activation/repression) of different genes in violacein pathway leads to production of different violacein derivatives (The figure is reprinted with permission from Elsevier: Zalatan *et al.* Engineering Complex Synthetic Transcriptional Programs with CRISPR RNA Scaffolds. *Cell* 2015;**160**:339–50.): VP64-activator domain, PP7/MS2 – RNA hairpin structures, PCP/MCP—RNA binding proteins. (**D**) Production of naringenin from glucose. Cas9-mediated one-step integration of the naringenin pathway into an intergenic locus. Downregulation of *TSC13* mediated by catalytically inactive (‘dead’) dCas9 (CRISPRi) to avoid the formation of by-products (The figure adapted from Vanegas, Lehka and Mortensen 2017).

CRISPR/Cas genome editing and transcriptional regulation are particularly suitable for developing yeast cell factories. As the strain development usually proceeds through iterative design-build-test cycles, the CRISPR technology facilitates this process because the strains can repeatedly be edited in a flexible multiplex way. As far as transcriptional regulation is concerned, CRISPR also enables relatively easy multiplexing. We will illustrate this with four brief examples (Fig. 4).

Shi *et al.* (2016) applied CRISPR/Cas9 to engineer *Saccharo-myces cerevisiae* towards production of a non-native product (*R*,*R*)-2,3-butanediol (BDO) from a non-native substrate xylose in a single transformation step. A 24-kb integration construct consisting of six gene expression cassettes (three for the xylose consumption pathway and three for the BDO biosynthesis pathway) was integrated into the delta sequence of the Ty transposon elements. Introducing Cas9-mediated DSBs at the delta sites allowed the integration of 10 copies of the 24-kb DNA fragment. A higher copy number of the pathways resulted in both higher xylose consumption rate and higher BDO production, where 0.31 g/L of BDO was produced from 20 g/L xylose (Shi *et al.*^2016^).

Stovicek *et al.* (2015) engineered diploid industrial *S. cerevisiae* strain Ethanol Red, used in many first generation ethanol plants, to produce lactic acid by replacing both alleles of pyruvate decarboxylase genes *PDC1* and *PDC5* with L-lactate dehydrogenase encoding gene (*ldhL*) from *Lactobacillus plantarum*. The genetic modification was accomplished in a single transformation event, leading to a strain producing 2.5 g/L lactic acid with the yield of 0.49 g of lactic acid/g of glucose (Stovicek, Borodina and Forster 2015).

Transcriptional regulation via CRISPRi was demonstrated for the production of the bacterial pigment violacein in *S. cerevisiae*. Here CRISPR RNA scaffolds were used to recruit transcriptional activators and repressors, alone or simultaneously, to a promoter site, which allowed tight control of transcriptional activation and repression. By simply changing the RNA scaffolds, the same strain could be reprogrammed to produce different ratios of the pathway products, deoxyviolacein, violacein, prodeoxyviolacein and proviolacein. Combining these RNA-encoded circuits with conditional expression of Cas9, a system for switching from growth to production phase was obtained (Zalatan *et al.*^2015^).

Recently, a combination of Cas9 genome editing and dCas9 transcriptional regulation was demonstrated by engineering *S. cerevisiae* for production of flavonoid precursor naringenin. First, Cas9 was used for integration of a multigene pathway into an intergenic locus leading to production of naringenin from phenylalanine. Next, the naringenin production was increased through dCas9-mediated downregulation of an essential gene *TSC13* to prevent the formation of by-product phloretic acid (Vanegas, Lehka and Mortensen 2017).

## OUTLOOK

This review summarizes the recent developments of CRISPR-based systems for genome editing and transcriptional regulation in various yeast species. The CRISPR/Cas9 technology has advantages over conventional marker-based genome editing in several aspects. It enables fast strain engineering of prototrophic wild and industrial yeast strains. Furthermore, it allows performing multiple genome edits simultaneously and is independent of marker cassette integration. For transcriptional regulation, the CRISPR offers an advantage of relatively easy design and implementation, the possibility of multiplexing and orthogonality. However, to enable the wide adaptation of CRISPR, the current limitations need to be addressed. These include (i) design of efficient and specific targeting for different yeast species, (ii) elimination of cloning necessity, (iii) enabling large-scale multiplexing and, finally, (iv) resolving the IP issues. The uncertainty about the ownership of the CRISPR technology delays its adaptation for industrial biotechnology and pharmaceutical applications and must be resolved as soon as possible so the technology can unfold its true potential.
